# Deep learning model for protein multi-label subcellular localization and function prediction based on multi-task collaborative training

**DOI:** 10.1093/bib/bbae568

**Published:** 2024-11-02

**Authors:** Peihao Bai, Guanghui Li, Jiawei Luo, Cheng Liang

**Affiliations:** School of Information and Software Engineering, East China Jiaotong University, No. 808 Shuanggang East Road, Nanchang 330013, China; School of Information and Software Engineering, East China Jiaotong University, No. 808 Shuanggang East Road, Nanchang 330013, China; College of Computer Science and Electronic Engineering, Hunan University, No. 2 Lushan Road, Changsha 410082, China; School of Information Science and Engineering, Shandong Normal University, No. 1 University Road, Jinan 250358, China; Shandong Key Laboratory of Biophysics, Dezhou University, No. 566 University Road, Dezhou 253023, China

**Keywords:** subcellular localization, protein function prediction, graph transformer, pre-trained language model, multi-task collaborative training

## Abstract

The functional study of proteins is a critical task in modern biology, playing a pivotal role in understanding the mechanisms of pathogenesis, developing new drugs, and discovering novel drug targets. However, existing computational models for subcellular localization face significant challenges, such as reliance on known Gene Ontology (GO) annotation databases or overlooking the relationship between GO annotations and subcellular localization. To address these issues, we propose DeepMTC, an end-to-end deep learning-based multi-task collaborative training model. DeepMTC integrates the interrelationship between subcellular localization and the functional annotation of proteins, leveraging multi-task collaborative training to eliminate dependence on known GO databases. This strategy gives DeepMTC a distinct advantage in predicting newly discovered proteins without prior functional annotations. First, DeepMTC leverages pre-trained language model with high accuracy to obtain the 3D structure and sequence features of proteins. Additionally, it employs a graph transformer module to encode protein sequence features, addressing the problem of long-range dependencies in graph neural networks. Finally, DeepMTC uses a functional cross-attention mechanism to efficiently combine upstream learned functional features to perform the subcellular localization task. The experimental results demonstrate that DeepMTC outperforms state-of-the-art models in both protein function prediction and subcellular localization. Moreover, interpretability experiments revealed that DeepMTC can accurately identify the key residues and functional domains of proteins, confirming its superior performance. The code and dataset of DeepMTC are freely available at https://github.com/ghli16/DeepMTC.

## Introduction

Proteins are crucial molecules in living organisms, which play key roles in biological processes such as signaling, gene regulation, substance transport, and biochemical catalysis [1–3]. Consequently, the study of protein function has become popular. This research has not only advanced our understanding of biological macrogenomics and pathogenic mechanisms [4, 5] but has also accelerated the discovery of new drug targets and the development of new drugs [6]. Current protein function studies encompass a range of approaches, including Gene Ontology (GO) annotation and subcellular localization. Many wet-lab methods are available for protein function studies. For example, protein function can be determined through biochemical assays and enzyme analyses, and subcellular localization can be identified via fluorescent biomarker tags [7]; however, these methods often require considerable time and costly equipment. Therefore, the emergence of computational methods is crucial and inevitable for large-scale protein function studies.

GO annotation contains three sub-ontologies: biological process (BP), cellular component (CC), and molecular function (MF). These existing methods can be categorized into four main types of protein function prediction: sequence-based protein function prediction, structure-based protein function prediction, protein–protein interaction (PPI)-based protein function prediction, and ensemble-based protein function prediction [8]. With respect to sequence-based protein function prediction models, the tools BLAST [9] and Diamond [10] were initially employed to functionally annotate target proteins. With the development of deep learning, many models have emerged that use deep learning to extract sequence features. DEEPred [11] uses deep neural networks (DNNs) for multi-group GO annotation prediction by stacking multiple layers of feed-forward DNNs. PANDA2 [12] uses a protein pre-trained language model to extract features of sequences and a graph neural network to combine different features to predict GO terms at different levels. ATGO [13] also employs a protein language model to extract sequence features and incorporates a comparative learning strategy into a triple network to extract potential protein functional features. With the emergence of tools such as AlphaFold2 [14], which enables accurate protein structure prediction, protein structure is beginning to be widely used in functional prediction tasks. For example, DeepFRI [15] utilizes graph convolutional neural networks (GNNs) to predict protein function, employs deep learning technology to extract residue-level protein features. TransFun [16] uses a transformer-based protein language model and equivariant graph neural network [17] to extract the feature information of proteins. Unlike the above two approaches, GAT-GO [18] uses a trained protein language model to extract sequence features, Raptor X [19] to predict the 3D structure of proteins. The PPI-based protein function prediction method [20–22] utilize the interaction information between proteins to annotate the protein functions. For example, DeepGO [20] uses 3-mers to encode the protein sequence, and DeepWalk [23] generates feature information of the protein in the PPI network. deepNF [21] utilizes the random walk with restart algorithm to learn feature embeddings of proteins on different heterogeneous PPI networks and uses an autoencoder to learn information about latent features of proteins. MSF-PFP [22] predicts protein function by combining multi-source protein feature information. Since then, developers have created ensemble prediction methods [24, 25] that leverage multi-source information and multiple predictors. GOLabeler [24] predicts protein features by integrating multiple sequence-based classifiers including Naive, BlastKNN, LR-3mer, LRInterPro, and LR-ProFET and improves prediction performance by integrating features from different methods using the learning-to-rank paradigm. DeepGraphGO [25] utilizes multi-species proteins to construct a large PPI network. The initial features of the nodes combine protein structural domain information and family information. GNNs are then used to update the node features.

However, these four methods have certain limitations as follows: (i) sequence-based protein function prediction methods ignore structural information, limiting the model’s ability to capture comprehensive protein details. (ii) Structure-based prediction methods often use graph neural networks to learn structural information; however, they suffer from over-smoothing and cannot address the long-range dependency problem. (iii) PPI network-based prediction methods rely heavily on known protein interactions, making them ineffective for predicting newly discovered proteins. (iv) Effective combination of different types of feature information remains a key challenge for ensemble-based prediction methods.

In recent years, computational approaches have made significant progress in determining the subcellular localization of proteins. Several researchers [26–28] have proposed using protein sequences for subcellular localization. SCLpred-EMS [26] employs an N-to-1 convolutional neural network to predict subcellular localization, by processing vector representations derived from homologous sequence comparison results. MULocDeep [27] utilizes two-layer bidirectional long short-term memory (LSTM) to process amino acid embeddings of protein sequences and the multi-head self-attention layer and LSTM output for context matrix derivation. DaDL-SChlo [28] uses a protein language model to learn protein sequence features and combines them with handcrafted features. In addition to the use of protein sequence information, several researchers have developed knowledge-based prediction models [29–31]. ML-locMLFE [29] adopts various feature extraction methods to obtain multi-source information, including pseudo amino acid composition, encoding on based the basis of grouped weights, GO and so on. ML-FGAT [30] extracts multi-source information, including sequence data, chemical–physical properties, evolutionary information, and structural information of a protein. GPSFun [31] is a multitask learning model that uses a high-precision large language model to predict structural information and extract sequence features, and finally utilizes the graph neural network to update protein features for prediction.

Although the aforementioned advanced computational models have made significant strides in subcellular localization tasks [32], they still possess the following shortcomings: (i) sequence-based computational models ignore the structural information of proteins; (ii) knowledge-based computational models rely heavily on known GO annotation databases, making them unable to predict newly discovered proteins with no known annotations; and (iii) structure-based computational models, although they combine sequence features and structural information, overlook the impact of GO annotations on protein subcellular localization.

To address the shortcomings in computational models for protein function prediction and subcellular localization, we propose DeepMTC, an end-to-end deep learning-based computational model, that employs a multi-task collaborative training strategy for three sub-ontology predictions and multi-label subcellular localization predictions. First, a pre-trained language model is employed to determine the 3D structure of the protein, and a protein language model is used to extract features from the protein sequence. A graph transformer (GT) is subsequently used to update the protein embedding features, and a multi-channel graph autoencoder captures various GO features of the proteins. Finally, self-attention pooling is applied to predict protein functions, and an attention mechanism integrates the learned protein functional features for multi-label subcellular localization of the predicted proteins.

## Materials and methods

### Datasets

The multi-label subcellular localization information of the proteins was downloaded from SwissProt and TrEMBL in the UniProt [33] database, and the GO annotations of the proteins were downloaded from EMBL-EBI [34]. First, we selected proteins from two species (human and mouse). Next, we focused on proteins whose amino acid sequences did not exceed 1200 amino acids. Finally, we co-screened the proteins for multi-label subcellular localization and GO annotations (selected GO annotations with a frequency of ≥20 occurrences and 10 subcellular localization labels). As a result, the dataset contains 6083 protein sequences and the corresponding GO annotations and multi-label subcellular localization, as shown in Tables S1 and S2. This dataset was randomly divided into a training set (80% of the dataset), a validation set (10% of the dataset), and a test set (10% of the dataset).

### Graph representation and feature processing

Protein function is closely associated with the interactions between atoms. However, computing the association maps between all atoms in a substantial amount of protein data is a time-consuming and resource-intensive undertaking. Therefore, we opted to use residues to construct 2D representations of proteins to study the interactions between residue pairs.

In this study, we used the pre-trained model ESM-Fold [35] to obtain a reliable 3D structure of the protein and constructed a contact map based on the relative distances ≤4.5 Å between alpha carbon atoms (Cα) in the 3D structure, calculated as $G=\left[\left\{V\right\},\left\{E\right\}\right]$. $\left\{V\right\}$ represents the set of nodes, the node features are initialized by the protein pre-training language model ESM-2 [35]. By referring to the original sequence lengths in the input FASTA file, we truncate the extracted features accordingly, thus maintaining consistency between the sequence features and the actual sequence amino acid lengths, and the features of each node are described as $h\in{R}^{d_h\times 1}$. $\left\{E\right\}$ denotes the set of edges in the residual contact map and we integrate information from multiple sources to initialize the following edge features: (i) Cα distances, (ii) sine and cosine encoding of the Cα coordinates, (iii) coordinates of the five nearest neighbors, and (iv) the sum of the neighbor distances, with the edge between node *i* and node *j* described as ${e}_{ij}\in{R}^{d_e\times 1}$.

### DeepMTC architecture overview

**Figure 1:**
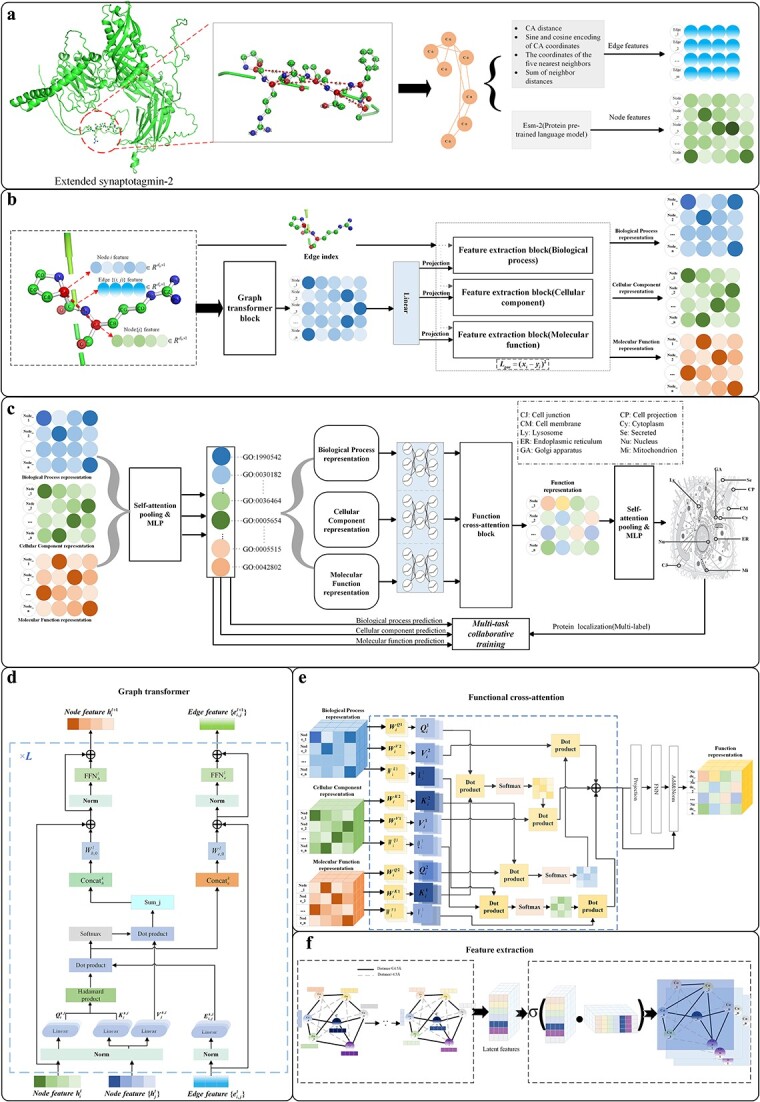
The overview of DeepMTC framework. (a) Acquisition of 3D protein structure and construction of residue contact maps, initialization of node features, and edge features of the contact map. (b) The node features and edge features of the residues are updated using the GT module and the functional features of the protein are learned with Gae_block. (c) Collaborative training: multi-task collaborative training strategy. (d) Architecture of the GT block, updating the node features and edge features of the residue contact graph. (e) Architecture of functional the cross-attention (Fun_attention) block for efficient combination of protein functional features. (f) Architecture of the feature extraction (Gae_block) block for extracting the functional features of proteins.

The overall process framework of DeepMTC is shown in Fig. 1. The protein is represented as 2D residue contact maps *G*, with residues as nodes and residue pair distances <4.5 Å as edges. Feature coding of proteins is performed by learning interactions between residue pairs.

To efficiently encode residues, we choose the GT block to update the residues and edges of proteins; the block captures residue interactions from the graph structure information, obtains long-range dependency associations and extracts global residue information of proteins. The graph autoencoder (Gae_block) is then used as the extraction module for the functional features.


(1) \begin{equation*} {H}_p^l,{E}_p^l= GT\left({H}_p^{\left(l-1\right)},{E}_p^{\left(l-1\right)}\right) \end{equation*}

(2) \begin{equation*} {H}_p^{bp}= Gae\_ bloc{k}_{bp}\left({W}_{bp}^2\left({W}_{bp}^1{H}_p^l+{b}_{bp}^1\right)+{b}_{bp}^2,\left\{E\right\}\right) \end{equation*}

Similarly, we use Equation (2) to obtain the functional features ${H}_p^{cc}$ and ${H}_p^{mf}$. Where *H* and *E_p_* represent the features of nodes and edges, respectively, and {*E*} denotes the edge index of the residue contact graph. The subscripts *p*, *bp*, *cc*, and *mf* represent protein, BP, CC, and MF, respectively.

By obtaining the features ${H}_p^{bp}$, ${H}_p^{cc}$, and ${H}_p^{mf}$ of different functional annotations of proteins, self-attention pooling [36] (SAT_pool block detailed process in Note 1 and Fig. S1) can be used to predict the three functional annotations of the most proteins.


(3) \begin{equation*} Scor{e}_{bp}= ML{P}_{bp}\left( SAT\_ poo{l}_{bp}\left({H}_p^{bp},\left\{E\right\}\right)\right) \end{equation*}

Meanwhile, we use Equation (3) to obtain the functional features $Scor{e}_{cc}$ and $Scor{e}_{mf}$. Multi-label subcellular localization is achieved by combining the three functional features of the learned proteins (Fun_attention block, Equation (4)). Instead of performing a search of a known GO annotation database to convert the GO annotations of proteins into feature representations, the model leverages the functional features of proteins learned upstream of the model for subcellular localization.


(4) \begin{equation*}\kern-.4pc {H}_p^{sl}= Fun\_ attention\left( nLinea{r}_{sl}^{bp}\left({H}_p^{bp}\right), nLinea{r}_{sl}^{cc}\left({H}_p^{cc}\right), nLinea{r}_{sl}^{mf}\left({H}_p^{mf}\right)\right) \end{equation*}

(5) \begin{equation*} Scor{e}_{sl}= ML{P}_{sl}\left( SAT\_ pool\left({H}_p^{sl},\left\{E\right\}\right)\right) \end{equation*}

where ${H}_p^{sl}$ represents node features and the *nLinear* (·) is a nonlinear transformation layer with two MLP layers. {*E*} denotes the edge index of the residue contact graph. We use a self-attention pooling layer to transform the protein feature representation into a vector representation, which is then passed through the MLP layer to obtain the subcellular localization scores for each protein class. We provide a succinct and systematic exposition of our proposed model, as shown in Algorithm S1.

### Graph transformer block

The GT combines the strengths of both GNNs and traditional transformer models, which leverages the graph’s topology for more effective learning of graph-structured data. Furthermore, the GT is computationally more efficient than the ordinary transformer. Instead of calculating attention scores between all possible pairs of nodes, it restricts these computations to node pairs connected by edges, as indicated by the graph edge index. Thus, the GT stands out as an exceptionally effective encoder for graph-structured data.

The node embedding ${h}_i\in{R}^{d_h\times 1} $ of the *i*th node and the edge embedding ${e}_{ij}\in{R}^{d_e\times 1} $ between nodes *i* and *j* are initialized to obtain the initial $ {h}_{p\_i}^0 $ and $ {e}_{p\_ ij}^0 $ with *d_p_* dimensions by leveraging two linear layers as follows:


(6) \begin{equation*} {h}_{p\_i}^0={W}_h^0{h}_i+{b}_h^0 \end{equation*}

(7) \begin{equation*} {e}_{p\_ ij}^0={W}_e^0{e}_{ij}+{b}_e^0 \end{equation*}

where ${W}_h^0\in{R}^{d_p\times{d}_h}$, and ${W}_e^0\in{R}^{d_p\times{d}_e}$ are learnable parameters in the linear layer, and ${b}_e^0$, ${b}_h^0\in{R}^{d_p}$ are learnable biases in the linear layer. The subscript *p* represents the protein. The feature update process for the *l*th layer of the GT is as follows:


(8) \begin{equation*} {Q}_{p\_i}^{k,l}={W}_Q^{k,l} Norm\left({h}_{p\_i}^l\right),\kern0.5em {K}_{p\_j}^{k,l}={W}_K^{k,l} Norm\left({h}_{p\_j}^l\right),\kern0.5em {V}_{p\_j}^{k,l}={W}_V^{k,l} Norm\left({h}_{p\_j}^l\right)\kern0.5em \end{equation*}

(9) \begin{equation*} {E}_{il}^{k,l}={W}_E^{k,l} Norm\left({e}_{p\_ ij}^l\right) \end{equation*}

(10) \begin{equation*} {w}_{p\_ ij}^{k,l}={\mathrm{softmax}}_{j\in N(i)}\left(\left(\frac{Q_{p\_i}^{k,l}\cdot{K}_{p\_j}^{k,l}}{\sqrt{d_k}}\right)\cdot{E}_{ij}^{k,l}\right) \end{equation*}

(11) \begin{equation*} {\left.{\overline{h}}_{p\_i}^l={h}_{p\_i}^l+{W}_{h,0}^l\Big( Concat\right|}_{k=1}^{h_d}\left\{ Aggregation\_ su{m}_{j\in N(i)}\left({w}_{p\_ ij}^{k,l}\cdot{V}_{p\_j}^{k,l}\right)\right\}\Big) \end{equation*}

(12) \begin{equation*} {\left.{\overline{e}}_{p\_ ij}^l={e_{p\_ ij}}_l+{W}_{e,0}^l\Big( Concat\right|}_{k=1}^{h_d}\left\{{w}_{p\_ ij}^{k,l}\right\}\Big) \end{equation*}

(13) \begin{equation*} {h}_{p\_i}^{l+1}={\overline{h}}_{p\_i}^l+{W}_{h2}^l\left( SiLU\left({W}_{h1}^l Norm\left({\overline{h}}_{p\_i}^l\right)\right)\right) \end{equation*}

(14) \begin{equation*} {e}_{p\_ ij}^{l+1}={\overline{e}}_{p\_ ij}^l+{W}_{e2}^l\left( SiLU\left({W}_{e1}^1 Norm\left({\overline{e}}_{p\_ ij}^l\right)\right)\right) \end{equation*}

where ${W}_Q^{k,l}$, ${W}_K^{k,l}$, ${W}_V^{k,l}$, ${W}_E^{k,l}\in{R}^{d_k\times{d}_p}$; ${W}_{h,0}^l$, ${W}_{e,0}^l\kern0.5em \in{R}^{d_p\times{d}_p}$; ${W}_{h1}^l$, ${W}_{e1}^1\in{R}^{2{d}_p\times{d}_p}$; and ${W}_{h2}^l$, ${W}_{e2}^l\in{R}^{d_p\times 2{d}_p}$ are learnable model parameter matrices. *Norm*(·) indicates batch normalization. *h_d_* and *d_k_* denote the number of heads and the feature dimension of each head of the multi-head attention mechanism, respectively. *SiLU* (·) denotes a type of activation function. ${\mathrm{softmax}}_{j\in N(i)}$ indicates a softmax operation on neighbor node *j* of node *i*. $Aggregation\_ su{m}_{j\in N(i)}$ denotes the summation of the messages on the edge consisting of node *i* and its neighboring node *j*. ${\left. Concat\right|}_{k=1}^{h_d}$ represents the output of concatenating multiple heads of the multi-head attention mechanism.

### Feature extraction block

In the protein function prediction task, Gae_block captures the intricacy of the relationships and interdependencies between nodes in a graph through the encoding and decoding process, which aids in the analysis of the graph structure and node relationships of the graph. The process of encoding the features is as follows:


(15) \begin{equation*} {H}_p^l=\left\{{h}_{p\_1},{h}_{p\_2},{h}_{p\_3},\cdots, {h}_{p\_n}\right\} \end{equation*}

(16) \begin{equation*} {H}_p^{bp}= Leaky\_\operatorname{Re} LU\left({\hat{D}}^{-0.5}\overline{A}{\hat{D}}^{-0.5}{H}_p^l{W}_{f\_ bp}^1\right) \end{equation*}

where *h* represents the node features, ${H}_p^l$ represents the node feature matrix, and $\overline{A}=A+I$ represents the adjacency matrix with self-loops. ${W}_{f\_ bp}^1\in{R}^{d_e\times{d}_{fe}}$ is a learnable parameter. $\hat{D}$ denotes the normalized diagonal matrix. The subscript *n* indicates the number of nodes in the contact graph for each protein. We perform decoding of features with inner products to reconstruct the adjacency matrix of the graph and use the MSE loss function [37] on the reconstructed adjacency matrix and the original computational loss to learn latent features.


(17) \begin{equation*} \hat{A}=\sigma \left({H}_p^{bp}\cdot{\left({H}_p^{bp}\right)}^T\right) \end{equation*}

(18) \begin{equation*} {L}_{bp\_ mse}=\frac{1}{n}\sum \limits_{i=1}^n{\left|{\overline{A}}_i-{\hat{A}}_i\right|}^2 \end{equation*}

where $\sigma \left(\cdot \right)$ denotes the activation function. ${L}_{bp\_ mse}$ indicates the value of the reconstructed loss. We repeat the learning process above to obtain the identity representations of the MF and CC as ${H}_p^{cc}$ and ${H}_p^{mf}\in{R}^{n\times{d}_{fe}}$, as well as their respective losses ${L}_{cc\_ mse}$ and ${L}_{mf\_ mse}$. ${d}_{fe}$ denotes the dimension of the feature.

### Functional cross-attention block

The Fun_attention block implements an adaptive and efficient combination of multiple functional annotation features of proteins, which include MF features ${H}_p^{mf}\in{R}^{n\times{d}_{fe}}$, CC features ${H}_p^{cc}\in{R}^{n\times{d}_{fe}}$, and BP features ${H}_p^{bp}\in{R}^{n\times{d}_{fe}}$. The Fun_attention block leverages multiple attention fusion mechanisms to adaptively compute the weights of different functional features of proteins, which can capture essential feature information to facilitate subcellular localization. The functional features use the multi-head attention mechanism as follows:


(19) \begin{align*} &{\left.{Q}_1^{k1},{Q}_2^{k2},{Q}_3^{k3}=\left({W}_{i,Q}^k{H}_p^{bp}\right)\right|}_{i=1}^3,\kern0.5em {K}_1^{k1},{K}_2^{k2},{K}_3^{k3}=\nonumber\\&\quad\left({W}_{i,K}^k{H}_p^{mf}\right){\left|{}_{i=1}^3,\kern0.5em {V}_1^{k1},{V}_2^{k2},{V}_3^{k3}=\left({W}_{i,V}^k{H}_p^{cc}\right)\right|}_{i=1}^3 \end{align*}

(20) \begin{equation*} {H}_p^1,{H}_p^2,{H}_p^3=\Big[ Concat{\left|{}_{ki=1}^{h_{ki}}\left(\mathrm{softmax}\left(\frac{Q_i^{ki}\cdot{K}_i^{ki}}{\sqrt{d_{ki}}}\right){\mathrm{V}}_i^{ki}\right)\Big]\right|}_{i=1}^3 \end{equation*}

(21) \begin{equation*} {H}_F=\psi \left({O}^t\cdot mean\left({H}_p^1,{H}_p^2,{H}_p^3\right)\right) \end{equation*}

(22) \begin{equation*} {H}_{SL}={H}_F+\psi \left({W}_F^2\left(\psi \left({W}_F^1{H}_F\right)\right)\right) \end{equation*}

where ${W}_{i,Q}^k\in{R}^{d_{ki}\times{d}_{fe}}$, ${W}_{i,Q}^k\in{R}^{d_{ki}\times{d}_{fe}}$, and ${W}_{i,Q}^k\in{R}^{d_{ki}\times{d}_{fe}}$ are learnable model parameters. $ki$ denotes the number of heads of the multi-head attention mechanism, and ${O}^t\in{R}^{d_F\times{d}_{fe}}$, ${W}_F^1\in{R}^{d_F\times 2{d}_F}$ and ${W}_F^2\in{R}^{2{d}_F\times{d}_F}$ are learnable parameters. ${d}_{k1},{d}_{k2}$ and ${d}_{k3}$ indicate the dimensions of each multi-attention mechanism, respectively. $Concat$ denotes the concatenation operation. $mean$ denotes the averaging operation for node features. $\psi \left(\cdot \right)$ represents an activation function (*ReLU*).

### Training protocol

In the training stage, DeepMTC applies a multi-task collaborative training model, using the three subtasks of protein function prediction as auxiliary tasks. The features learned from the upstream protein function prediction task were utilized in the subcellular localization of proteins. Therefore, the loss of function prediction was considered as part of the overall loss during training, and DeepMTC simultaneously supervises multi-task collaborative training. We first calculate the losses for the three subtasks of predicting protein function as ${L}_{bp\_ bce}$, ${L}_{cc\_ bce}$, and ${L}_{mf\_ bce}$. In learning the functional features of proteins, the Gae_block employs a graph autoencoder to update the graph structure and uses ${L}_{bp\_ mse}$, ${L}_{cc\_ mse}$, and ${L}_{mf\_ mse}$ to calculate the loss for the functional prediction subtask:


(23) \begin{equation*} {L}_{bp\_ bce}=\frac{1}{N\times M{}^{bp}}\sum \limits_{i=1}^N\sum \limits_{j=1}^{M^{bp}}\left(-{p}_{ij}^{bp}\log \left({\hat{p}}_{ij}^{bp}\right)-\left(1-{p}_{ij}^{bp}\right)\log \left(1-{\hat{p}}_{ij}^{bp}\right)\right) \end{equation*}

(24) \begin{equation*} {L}_{fun}={L}_{bp\_ bce}+{L}_{cc\_ bce}+{L}_{mf\_ bce} \end{equation*}

(25) \begin{equation*} {L}_{mse}={L}_{bp\_ mse}+{L}_{cc\_ mse}+{L}_{mf\_ mse} \end{equation*}

where *N* and *M* denote the number of proteins and the kinds of GO terms used for function prediction, respectively. *p* and $\hat{p}$ denote the true and predicted labels, respectively. ${L}_{fun}$ and ${L}_{mse}$ denote the overall loss of each of the function prediction and feature extraction modules.


(26) \begin{equation*} {L}_{sl}=\frac{1}{N\times C}\sum \limits_{i=1}^N\sum \limits_{j=1}^C\left(-{p}_{ij}^{sl}\log \left({\hat{p}}_{ij}^{sl}\right)-\left(1-{p}_{ij}^{sl}\right)\log \left(1-{\hat{p}}_{ij}^{sl}\right)\right) \end{equation*}

(27) \begin{equation*} {L}_{DeepMTC}=\alpha \cdot{L}_{fun}+\beta \cdot{L}_{mse}+\omega \cdot{L}_{sl} \end{equation*}

where *C* denotes the number of categories for subcellular localization. ${L}_{sl}$ and ${L}_{DeepMTC}$ indicate the loss of protein subcellular localization and the overall collaborative training loss of the DeepMTC model, respectively. $\alpha$, $\beta$ and $\omega$ are hyperparameters that determine the share of individual tasks in the collaborative training total loss.

## Results and discussion

### Experimental settings and evaluation metrics

In this study, DeepMTC is implemented using PyTorch on an Nvidia RTX 3090Ti GPU, which is trained using the Adam [38] optimizer with a learning rate of 0.0001. The training process uses alternating training and validation sets to search for effective model parameters, and finally an independent test set is used to evaluate the effectiveness of the model parameters, with the number of epochs set to 50. We fine-tune the model to identify the appropriate hyperparameters for superior performance; the critical parameter settings are shown in Table S3. Moreover, to comprehensively evaluate the performance of DeepMTC, we select a variety of metrics to evaluate the model from different perspectives. Details of the metrics are shown in Note 2.

### Performance of protein function prediction and subcellular localization on DeepMTC

**Figure 2:**
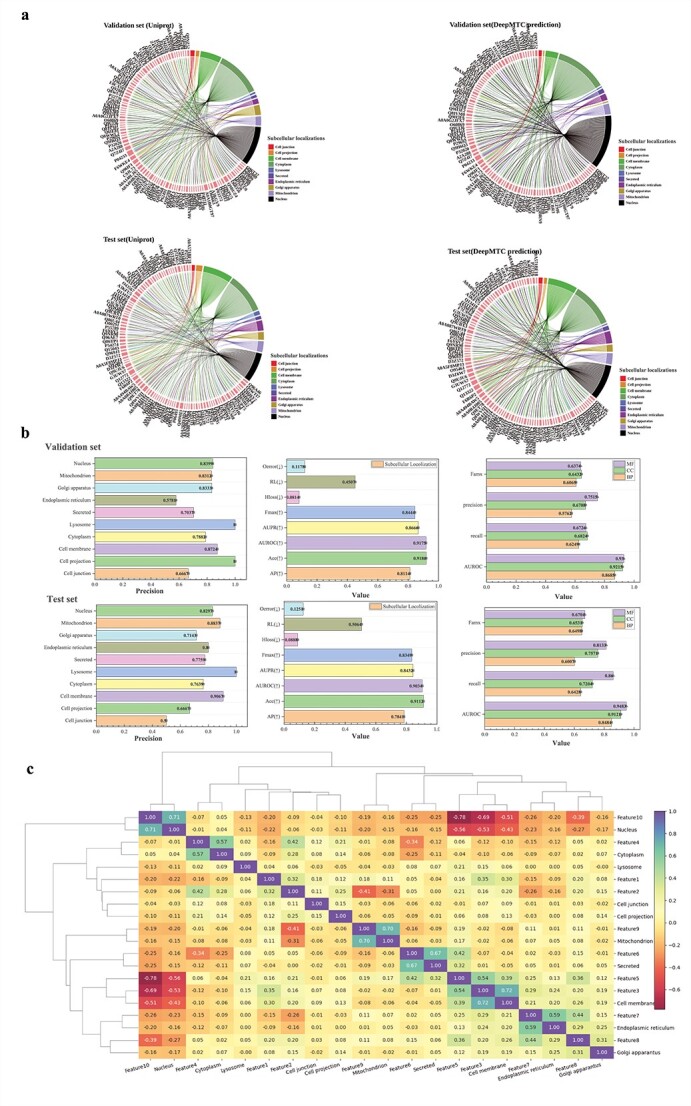
Performance of DeepMTC on an independent test set. (a) The predictions for 100 randomly selected samples from both the validation and test sets were compared with the labels in the UniProt database. (b) Performance of DeepMTC on the independent test set and on the validation set. (c) Correlation analysis between protein subcellular localization labels and features learned by DeepMTC.

We test the performance of DeepMTC on both the validation and independent test sets. For the protein subcellular localization task as shown in Fig. 2a, we can directly compare the labels predicted by DeepMTC with the corresponding subcellular localization of proteins in the UniProt database on the validation set and independent test set. A more detailed discussion of the results can be found in Note 3. DeepMTC achieves AP, Acc, and AUROC values on the validation set and test set of 81.14%, 78.41%, 91.86%, and 91.75%, 91.12%, 90.34%, respectively as shown in Fig. 2b. With respect to protein function prediction, the AUROC is >80% for all three sub-ontology predictions, and >90% for both BP and CC. We use a heatmap to represent the correlation between protein features learned by DeepMTC and protein subcellular localization labels as shown in Fig. 2c. The results reveal correlations between labels, as well as between features and labels, aiding in the investigation of relationships between different subcellular localizations. We analyse the relevance of labels in detail in Note 4.

However, to effectively demonstrate the superiority of DeepMTC, we compare it with state-of-the-art models. For a fair evaluation, we process the independent test set by filtering the localizations common to all three models, resulting in a new independent test set containing cell membrane, cytoplasm, lysosome, endoplasmic reticulum, Golgi apparatus, mitochondrion and Nucleus. We directly employ state-of-the-art models from the original publications, applying them to the new independent test set (seven localizations). State-of-the-art protein subcellular localization tools DeepLoc 2.0 [39] and GPSFun [31] for multi-label prediction (details shown in Note 5).

**Figure 3:**
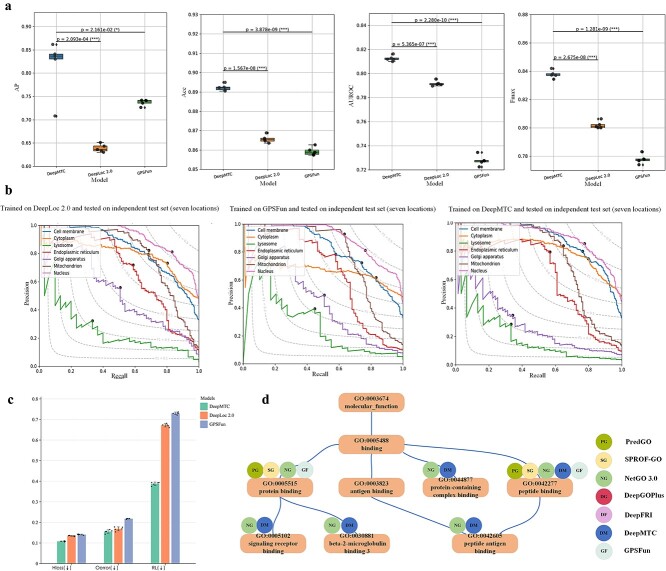
Performance comparison with existing methods and tools on the new independent test set. (a) Significance analysis of the four metrics—AP, Acc, AUROC, and Fmax—between DeepMTC and state-of-the-art models on the subcellular localization task. (b) Precision-recall curves showing the different methods at each location. (c) Comparison of DeepMTC and state-of-the-art methods on three metrics: Hloss (↓), RL and Oerror (↓). (d) DAG diagram of correctly predicted MF terms of P01899 using different methods.

The experimental results on the new independent test set are shown in Table 1, DeepMTC outperforms of DeepLoc 2.0 and GPSFun, and the values of the three metrics, Hloss, RL, and Oerror are lower than those of the other tools. To determine the performance difference between the models, the new independent test set was selected five times, with 80% of the samples randomly chosen each time. These five sets of data are then subjected to experiments as shown in Fig. 3a and c. The differences in the AP, Acc, AUROC, and Fmax values for DeepMTC compared with DeepLoc 2.0 and GPSFun are significant. To compare the performance of these tools more comprehensively, we plot the precision-recall curves for each model across all subcellular localizations, as shown in Fig. 3b. The results demonstrate that DeepMTC outperforms the other two tools in the cell membrane, cytoplasm, endoplasmic reticulum, mitochondrion, and nucleus. We analyse the reasons for the outstanding performance of DeepMTC in detail in Note 6.

**Table 1:** Performance comparison of DeepMTC with state-of-the-art methods on the subcellular localization task (independent test set with seven locations)

Method	AP	AUROC	AUPR	Acc	Fmax	Hloss (↓)	RL (↓)	Oerror (↓)
DeepLoc 2.0	0.6384	0.7916	0.7879	0.8655	0.8018	0.1344	0.6716	0.1688
GPSFun	0.7369	0.7278	0.7635	0.8593	0.7780	0.1407	0.7298	0.2175
DeepMTC	0.8426	0.8124	0.8415	0.8922	0.8377	0.1078	0.3878	0.1556

For the protein function prediction task, we use P01899 as an example to compare DeepMTC with other state-of-the-art models. The PredGO [40], SPROF-GO [41], NetGO 3.0 [42], DeepGOPlus [43], DeepFRI [15], and GPSFun [31] models are selected for this comparison. The methods are described in detail in Note 7. The primary goal of DeepMTC is to predict subcellular localization of proteins, with protein function prediction as a secondary task. Therefore, our independently constructed test set cannot be directly compared with existing functional prediction models. To address this, we use P01899 as an example in the experiments to compare the prediction performance of DeepMTC with that of other state-of-the-art models. We utilize other state-of-the-art models trained in the original publication for our experiments in the new test set. In Fig. 3d, the DAG plot shows the BP terms of P01899 based on the dataset in this study and the results correctly predicted by different methods. In Table S4, the correct and incorrect BP terms predicted by different methods are shown, and in Note 8, we analyse the case study of P01899 in detail.

### Impact of multi-task collaborative training strategy

**Figure 4:**
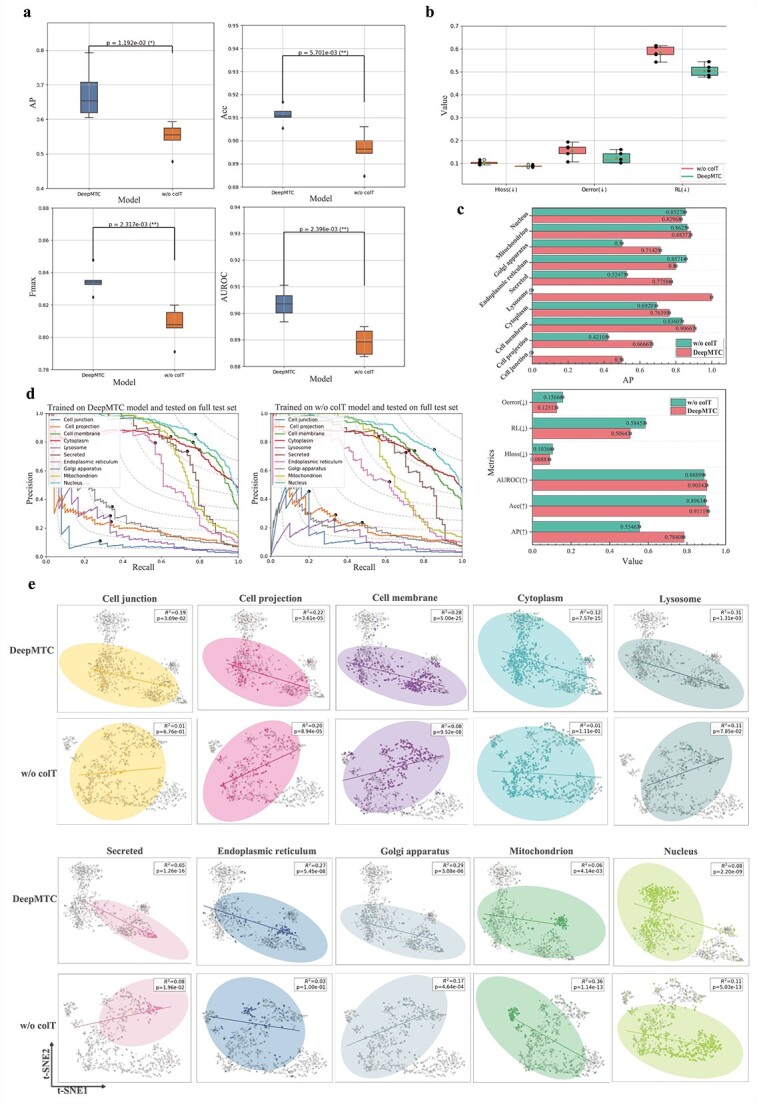
Multi-task collaborative training strategy effectively improves model performance. (a and b) Performance and minimization metrics comparison of DeepMTC and the model without the collaborative training strategy. Significance was tested using *t*-test. *P* values <.05, .01, and .001 are denoted by one to three asterisks, respectively. (c) DeepMTC achieves superior performance on the independent test set. (d) Precision-recall curves showing the different strategies at each location. (e) Feature visualization and correlation analysis (t-SNE algorithm).

During model training, it is widely believed that a single task can ease the overall task of the model, thus allowing for superior performance in model training. In this section, we explore the impact of multi-task collaborative training on model performance. We modify DeepMTC by removing the multi-task collaborative training strategy, creating a variant referred to as w/o colT. We train the two models separately on the training set and conduct five experiments on an independent test set, with each experiment randomly using 80% of the samples in the test set. The results are shown in Fig. 4a and b. Without the multi-task collaborative training strategy, the performance of the models on the subcellular localization task is significantly decreased, with statistically significant differences in model performance. On the independent test set, DeepMTC outperforms colT across all the metrics, as shown in Fig. 4c. DeepMTC achieves an AP of 78.40%, which is 23% higher than that of colT. Additionally, the minimization metrics Hloss, RL, and Oerror for DeepMTC exceed 90%.

We further explore the effect of the multi-task collaborative training strategy on the subcellular localization of each class separately. As shown in Fig. 4d. DeepMTC outperforms w/o colT in all seven localizations: cell junction, cell membrane, cytoplasm, mitochondrion, secreted, endoplasmic reticulum, and Golgi apparatus. These results correspond with those shown in Fig. 4c (upper panel), which show that DeepMTC consistently demonstrates higher performance across these localizations. Moreover, we visualized the protein features learned by DeepMTC and colT using t-SNE, as shown in Fig. 4e. We find that the coefficients of determination (R^2^) of DeepMTC are much greater than those of w/o colT in the six localizations of cell junctions, cell membrane, cytoplasm, secreted, endoplasmic reticulum, and Golgi apparatus, which indicates a greater degree of interpretation among the features learned by DeepMTC. In Fig. 4e (nucleus), the protein features learned by DeepMTC are distinctly divided into two regions based on their association with the nucleus, clearly showing a separation between nuclear-associated and non-nuclear-associated features.

### Ablation experiments

To assess the impact of the various modules of DeepMTC on the performance of the model, we conduct ablation experiments. We design three variants of the DeepMTC model:

- DeepMTC w/o GT removes the GT feature learning module.
- DeepMTC w/o FunA removes the functional across-attention module.
- DeepMTC w/o FeaE removes the functional feature extraction module.

**Figure 5:**
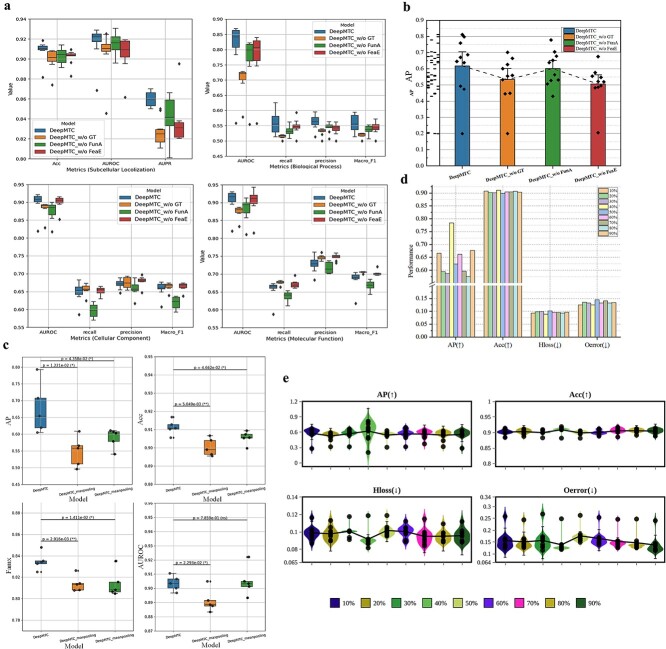
Ablation study of DeepMTC. (a) Performance comparison of DeepMTC and its variants on the validation set, with 10 experiments on a randomly selected 80% of the validation set. (b) Comparison of the AP of DeepMTC and its three variant models in the validation set. (c) Performance impact of different pooling strategies on model performance. (d and e) The impact of the pooling ratio of the node masking strategy in self-attention pooling algorithms on model performance.

The performances of DeepMTC and its variant models on the validation set for multitasking are shown in Fig. 5a and b. We find that removing the GT module had the most significant impact on the performance of the model in the subcellular localization task and the BP annotation prediction task. This result may be because the number of BP annotations in our dataset is relatively low, necessitating the GT module to extract more protein information to compensate for the lack of BP annotations. Additionally, the results for protein function prediction and subcellular localization on the independent test set are summarized in Tables S5Table S6. The GT module has the greatest impact on the performance of the model, which is consistent with our design, which uses the GT module as a core component.

To explore the impact of different pooling strategies on the task of protein subcellular localization prediction, we compare the results of self-attention pooling with those of max pooling and mean pooling, as shown in Fig. 5c, and we analyse the effect of pooling rate on the performance of the model as shown in Fig. 5d and e. We discuss in detail the impact of different pooling strategies and pooling rates on the model in Note 9. Additionally, we established a threshold composition based on the distance between the Cα during the construction of the residual maps. We then examined the impact of varying threshold levels on the model’s performance, with the experimental results presented in Tables S7 and S8. A detailed discussion of these results is provided in Supplementary Note 10.

### Gene Ontology enrichment analysis

**Figure 6:**
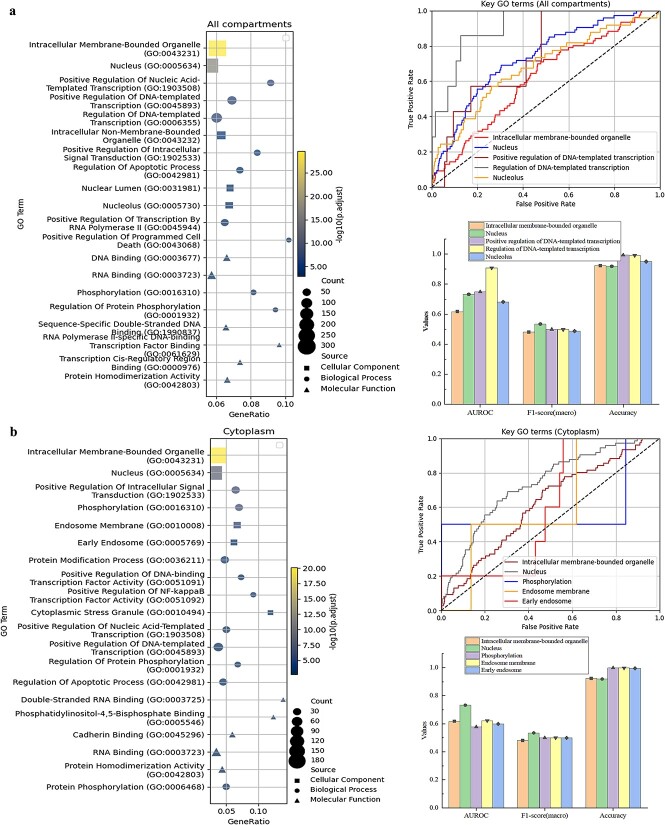
The top 20 enrichment terms for proteins localized to all compartments and Cytoplasm, and DeepMTC predictions. (a and b) The left panel shows a bubble plot of the top 20 GO terms for all the compartments and Cypolasm. The right panel presents DeepMTC predictions for these key terms. The accompanying ROC curves and bar charts highlight the model’s superior predictive performance for these terms.

To explore the correlation between the subcellular localization of proteins and their functions, we perform GO enrichment analysis on proteins to analyse their BPs, CCs, and MFs. We perform GO enrichment analysis separately for all proteins localized in the 10 compartments (All-ten) and for proteins localized in Cytoplasm, respectively as shown in Fig. 6. Due to space limitations, we will discuss the GO enrichment results and the DeepMTC predictions for key GO terms in Note 11.

### Interpretation of models by residue attention visualization

**Figure 7:**
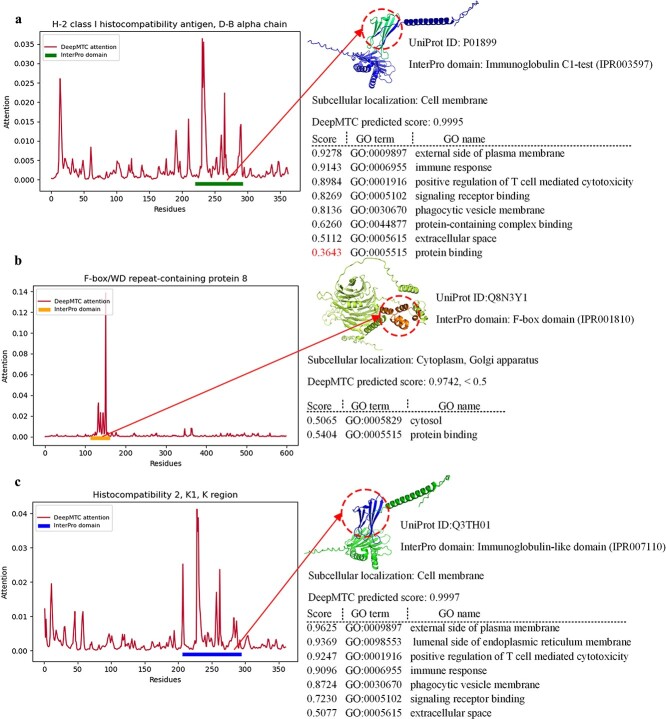
Interpretability of DeepMTC models by attention scores of residues. (a–c) Visualization of MF attention scores for three test set samples (UniProt ID: P01899, Q8N3Y1, and Q3TH01).

In previous experiments, we evaluated the performance of the model to determine the effectiveness of DeepMTC. However, it remains unclear whether the results of the model on the validation set proteins are solely based on the proteins from the training set or if it genuinely learns the deep biological properties of the proteins. To clearly explain the decision-making mechanism DeepMTC uses for prediction, we select three proteins from the MF prediction (UniProt ID): P10899, Q8N3Y1, and Q3TH01. We then extract the importance scores of the corresponding sequence residues as learned by DeepMTC. Simultaneously, we use InterProScan to search for InterPro domains corresponding to the sequences in the InterPro database [44]. We use the functional domains identified by InterProScan and the residue importance scores learned by DeepMTC to validate the decision-making mechanism of DeepMTC. As shown in Fig. 7a, the H-2 class I histocompatibility antigen, D-B alpha chain residue importance scores and corresponding InterPro domains are visualized on the left. P01899 contains an Immunoglobulin C1-test domain [45], which ranges from residues 220–293 in the sequence, and residues in this range have high importance scores. The protein has six GO terms, and DeepMTC successfully predicts five of them, achieving the highest prediction score of 92.94%. F-box/WD repeat-containing protein 8 (Q8N3Y1) contains an F-box domain [46] at residue positions 113–162 in the sequence, and residues within this range have the highest importance scores as shown in Fig. 7b. The F-box domain associates the protein with ‘protein binding’, and DeepMTC correctly predicts this term. Another case is Histocompatibility 2, K1, K region (Q3TH01), which contains an Immunoglobulin-like (Ig-like) domain [47]. The position of this InterPro domain in the sequence residues is 206–294, and residues in this range also have a high importance score, as shown in Fig. 7c. The Ig-like domain is involved in a variety of functions, including cell recognition and interaction with cell surface receptors [48]. This association links the protein to terms such as ‘peptide antigen binding’ and ‘signaling receptor binding’. DeepMTC correctly predicts both terms, with the prediction probability for ‘peptide antigen binding’ reaching 92.33%. By studying the importance of residues and sequence functional domains, we can conclude that DeepMTC not only performs exceptionally well in predicting protein functions and subcellular localization tasks but also excels in learning the biological properties of proteins. Therefore, it has outstanding accuracy in identifying key residues and functional domains.

## Discussion

The prediction of the subcellular localization of proteins is typically influenced by their functional information. Considering both factors together not only enhances model performance but also allows the model to learn more comprehensive information about the proteins. However, most existing methods for predicting subcellular localization suffer from low accuracy or require additional costs to search known databases, and some overlook the relationship between GO annotations and subcellular localization. Therefore, we propose DeepMTC, a model that fundamentally integrates subcellular localization with protein functional information while bypassing the need for expensive database searches. DeepMTC offers the following five remarkable features: (i) DeepMTC combines multi-source information, including sequence features, structural information, and GO, without requiring searches of known databases for GO annotations; (ii) it combines GT and graph auto-encoder techniques to deeply mine protein structural information; (iii) it uses a functional cross-attention module to efficiently combine functional features; (iv) it employs a multi-task collaborative training strategy to achieve excellent performance in both protein function prediction and subcellular localization tasks; and (v) it utilizes self-attention pooling to adaptively obtain protein embeddings, enhancing model interpretability.

However, DeepMTC also has limitations and room for further improvement. Our model relies on accurate 3D structures of proteins, as the results generated by the large language model directly impact the performance of DeepMTC. In the future, we plan to use only the 3D structures of proteins during the training phase to maximize the efficiency of model testing. Additionally, we will incorporate PPIs into the model to integrate information on protein interactions, facilitating a more comprehensive study of protein function. In future GO annotation prediction tasks, we will focus on less frequent and more challenging annotations to increase the model’s generalizability. In future studies, we aim to design a generalized computational model that integrates functional prediction, subcellular localization, ligand binding sites, solubility, and protein dynamics for a more in-depth analysis of proteins.

**Key Points**

- DeepMTC is a fully end-to-end deep learning approach that employs a multi-task collaborative training strategy to simultaneously and efficiently predict protein function and multi-label subcellular localization.
- DeepMTC employs a functional cross-attention module to efficiently combine protein functional features, enhancing prediction performance.
- DeepMTC uses GO features for predicting multi-label subcellular localization, without relying on established GO annotation databases.

## Supplementary Material

Supplementary_Information_bbae568

## Data Availability

The code and dataset of DeepMTC are freely available at https://github.com/ghli16/DeepMTC.
