# Supplementary material for: Deep learning model for protein multi-label subcellular localization and function prediction based on multi-task collaborative training
Source: Brief Bioinform. 2024 Nov 2;25(6):bbae568. doi: 10.1093/bib/bbae568 (PMC11531862; doi:10.1093/bib/bbae568)
Supplement: Supplementary_Information_bbae568 [file supplementary_information_bbae568.docx]

**Supplementary Information**

**Deep learning model for protein multi-label subcellular localization and function prediction based on** **multi-task** **collaborative training**

Peihao Bai^a^, Guanghui Li^a,*^, Jiawei Luo^b^, Cheng Liang^c,d,*^

^a^ School of Information and Software Engineering, East China Jiaotong University, Nanchang, 330013, China

^b^ College of Computer Science and Electronic Engineering, Hunan University, Changsha, 410082, China

^c^ School of Information Science and Engineering, Shandong Normal University, Jinan, 250358, China

^d^ Shandong Key Laboratory of Biophysics, Dezhou University, Dezhou, 253023, China

*Corresponding author: Guanghui Li, School of Information and Software Engineering, East China Jiaotong University, Nanchang, 330013, China. E-mail: ghli16@hnu.edu.cn; Cheng Liang, School of Information Science and Engineering, Shandong Normal University, Jinan, 250358, China. E-mail: alcs417@sdnu.edu.cn

**Supplementary Notes**

**Note 1**: Self-attention pooling.

**Note 2**: Detailed explanation of evaluation metrics.

**Note 3**: Further analysis - DeepMTC performance on multilabel subcellular localization task.

**Note 4**: Further analysis - Correlation analysis of labels.

**Note 5**: Details of the state-of-the-art protein multi-label subcellular localization tools DeepLoc 2.0 and GPSFun.

**Note 6**: Further analysis - Analysis of the reasons why different methods cause such experimental results.

**Note 7**: A detailed description of state-of-the-art protein function prediction methods.

**Note 8**: Further analysis - Different methods are analyzed based on P01899 functional prediction results.

**Note 9:** Further analysis - Impact of different pooling strategies and pooling rates on the model.

**Note 10:** Further analysis - Impact of different distance thresholds on the model.

**Note 11:** Further analysis - Analysis of GO enrichment results and prediction of key terms.

**Supplementary Tables**

**Table S1:** Descriptive statistics of two species.

**Table S2:** Subcellular localization and distribution of proteins.

**Table S3:** Hyperparameter setting of DeepMTC.

**Table S4:** Predicted GO terms of P01899 in MF by DeepMTC and competing methods.

**Table S5:** The ablation experiment result on the subcellular localization task.

**Table S6:** The ablation experiment result on the functional prediction task.

**Table S7.** The impact of residue map construction threshold on the subcellular localization task.

**Table S8.** The impact of residue map construction threshold on the functional prediction task.

**Supplementary Algorithm**

**Algorithm S1:** Algorithm of DeepMTC.

**Supplementary Notes**

**Note 1:** Self-attention pooling.

The SAT_pool block (**Figure S1**) conducts graph feature pooling based on the respective weights of the nodes in the graph, considering both the local properties of the nodes and the global message of the graph. We utilize a graph convolutional network [1] to compute the weight of each node based on the edge index *G*={*E*} and the node feature matrix$H\in R^{n\times d}$of the graph, and perform the pooling operation of the graph features based on the weight of the nodes, as shown in the following equations:

$$Soc=\sigma(\hat{D}^{-\frac{1}{2}}G\hat{D}^{-\frac{1}{2}}H\Theta_{atten})$$

$$\bar{H}=H\otimes Mask(Soc)$$

$$\hat{H}=Concat(ave\_pool(\bar{H}),\text{ }max\_pool(\bar{H}))$$

where $\sigma$ denotes an activate function (*tanh*), and$\Theta\in R^{d\times1}$ is a learnable parameter. $\hat{D}\in R^{n\times n}$ represents the degree matrix of the nodes in the graph. is the broadcasted elementwise product. *Mask* represents the attention mask, which enhances the generalization power and robustness of the model. $ave\_pool$, $max\_pool$and *Concat* denote the average pooling, max pooling, and concatenation operations, respectively.


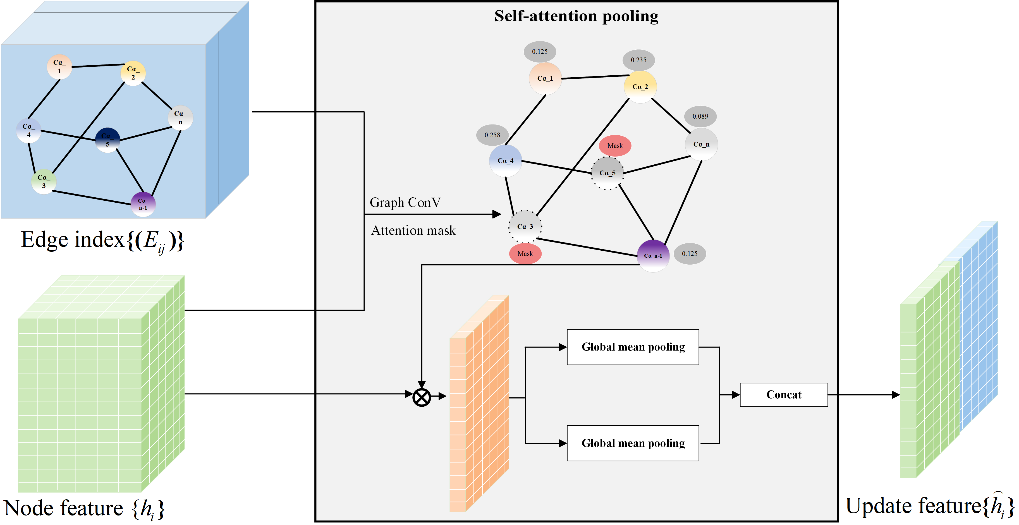


**Figure S1.** Flowchart for self-attention pooling.

**Note 2:** Detailed explanation of evaluation metrics.

The protein function prediction task is evaluated by five metrics: area under the receiver operating characteristic curve (AUROC), recall, precision, Macro_F1 and Fmax. The first four metrics are common task evaluation metrics used in studies by Davis et al [2], Clark and Radivojac [3] and Jiang et al [4]. The fifth metric is recommended by the Critical Assessment of protein Function Annotation (CAFA) [4] as a common protein function prediction evaluation metric used by Clark and Radivojac [3]. In addition, for the multi-label subcellular localization task, we used a combination of eight metrics, average precision (AP), accuracy (Acc), AUROC, area under the precision-recall curve (AUPR), Fmax, hamming loss (Hloss), ranking loss (RL), and One-error (Oerror), to provide a comprehensive and holistic evaluation of the model performance. These evaluation metrics for multi-label data refer to Zhang and Zhou [5]. These metrics encompass two aspects of sample-based and label-based metrics, which can be described in detail as four aspects of metrics based on sample classification and ranking, and label-based classification and ranking. Using these metrics, we can comprehensively measure the combined performance of DeepMTC.

**Note 3:** Further analysis- DeepMTC performance on multilabel subcellular localization task.

For example, the subcellular localizations of the HLA class II histocompatibility antigen gamma chain (P04233) listed in the UniProt database include Cell membrane, Endoplasmic reticulum, Golgi apparatus, Lysosome, Secreted and Cytoplasm. The predicted scores of DeepMTC for the seven localizations of the HLA class II histocompatibility antigen gamma chain are 0.3929, 0.9746, 0.4652, 0.7009, 0.9999 and 0.9852, respectively, and the prediction scores for the remaining localizations are all less than 0.1.

**Note 4:** Further analysis-correlation analysis of labels.

We observe a negative correlation between the nucleus and other organelles and a positive correlation between nucleus and cytoplasm, because some proteins are synthesized or activated in the cytoplasm and then move to the nucleus for gene expression and regulation. For example, the STAT, β-catenin, and FOXO transcription factors are proteins that transition between the cytoplasm and the nucleus to dynamically control gene expression in response to various internal and external environmental signals [6-8].

**Note 5:** Details of the state-of-the-art protein multi-label subcellular localization tools DeepLoc 2.0 and GPSFun.

- DeepLoc 2.0 [9] (2022): which encodes the input amino acid sequence using a converter-based protein language model. Interpretable attention pooling mechanisms are then used to generate sequence representations for the task of predicting subcellular localization.
- GPSFun [10] (2024): which employs a large language model to extract sequence embeddings. Then, a graph neural networks are also utilized to capture protein structural information for downstream multi-task prediction including subcellular localization.

**Note 6**: Analysis of the reasons why different methods cause such experimental results.

In order to understand the experimental results at a deeper level, we discuss the reasons for such results in terms of the nature of each model. DeepLoc 2.0 uses protein language models to extract protein sequence features but ignores the structural information of proteins. GPSFun combines protein language models with structural information to learn features; however, it does not leverage protein functional annotations, which are closely related to subcellular localization. DeepMTC combines the advantages of these models while addressing their limitations by utilizing protein topology and fully incorporating protein functional features. Experiments demonstrate that DeepMTC exhibits superior performance and is a highly robust and effective tool for protein subcellular localization.

**Note 7**: A detailed description of state-of-the-art protein function prediction methods.

- PredGO [11]: which utilizes pre-trained language models with geometric vector perceptrons and attention mechanisms to learn protein features, and ultimately combines these features to predict protein function.
- SPROF-GO [12]: which uses a pre-trained protein language model to extract amino acid sequence features and self-attention pooling to identify key residues.
- NetGO 3.0 [13]: which uses a protein language model (ESM-1b) to extract the features of each protein sequence and improves the performance of the model by integrating information from multiple sources using logistic regression for training.
- DeepGOPlus [14]: which combines a deep convolutional neural network (CNN) with sequence similarity-based prediction, relies on amino acid sequences to predict protein function.
- DeepFRI [15]: which utilizes protein language models and protein structures to extract sequence features of proteins and combines these features through graph convolutional networks for predicting protein functions.

**Note 8**: Further analysis - Different methods are analyzed based on P01899 functional prediction results.

In the case study of P01899, the following results were obtained and analysed using different methods of experimentation. DeepMTC and NetGO 3.0 correctly predict the most BP terms. However, NetGO 3.0 incorrectly predict five BP terms, whereas DeepMTC does not predict any BP terms incorrectly. In contrast, DeepGOPlus and DeepFRI do not predict any correct BP terms, and PredGO and SPROF-GO each correctly predict two BP terms. To summarize, DeepMTC uses a graph transformer to extract residue features and a graph autoencoder to learn the latent functional features of proteins. Additionally, it employs a multi-task collaborative training strategy, which achieves subcellular localization to significantly improve the accuracy and correctness of protein function prediction.

**Note 9**: Further analysis - Impact of different pooling strategies and pooling rates on the model.

To explore the impact of different pooling strategies on the task of protein subcellular localization prediction, we compare the results of self-attention pooling with those of max pooling and mean pooling, as shown in **Figure 5**C. We find that self-attention pooling significantly outperforms traditional pooling methods. Traditional pooling approaches ignore the variability among nodes and do not utilize graph topology for pooling in graph-level tasks. In contrast, self-attention pooling considers the variability of graph nodes and leverages graph topology for pooling operations. This effectively enhances the robustness and reliability of the pooling task at the graph level. We also explore the effects of different pooling ratios on model performance in predicting subcellular localization using self-attention pooling. The experimental results for the subcellular localization task on the independent test set are shown in **Figure 5**D. We find that the AP and Acc of the subcellular localization task are highest when the pooling ratio k = 40%, and the minimization metrics Hloss and Oerror are both the lowest. The ten experiments conducted on the validation set, shown in **Figure 5**E, are consistent with the results on the independent test set. Therefore, adopting self-attention pooling strategy and optimizing the pooling rate for the protein subcellular localization task can make DeepMTC more robust and generalizable.

**Note 10**: Further analysis - Impact of different distance thresholds on the model.

In constructing residue contact maps using the 3D structure of proteins, we define two residues as associated if the distance between their α-carbons (Cα) is less than a specified threshold. This thresholding approach allows us to effectively capture and characterize the interactions and spatial relationships between residues. Given that different thresholds can highlight varying interactions, we investigate the impact of distance thresholds on model performance by evaluating multiple values. Previous studies [16] and [17] utilized thresholds of 8 Å and 10 Å, respectively. To capture a more comprehensive range of amino acid interactions, we introduced a threshold of 4.5 Å for our experiments. The results are presented in **Table S7** and **S8**. In the subcellular localization task, we observed that the average precision (AP) value is significantly higher when using a threshold of 4.5 Å compared to the 8 Å and 10 Å thresholds. Similarly, in the functional prediction task, the AUROC value for 4.5 Å exceeds that of the other two thresholds across all three subtasks. Overall, the performance comparisons indicate that selecting 4.5 Å as the distance threshold for constructing residue maps allows for a more comprehensive capture of atomic interactions, providing detailed structural information that benefits downstream protein tasks.

**Note 11**: Further analysis - Analysis of GO enrichment results and prediction of key terms.

As shown in **Figure 6**A (left panel), the analysis reveals significant enrichment of the GO terms 'intracellular membrane-bounded organelle', 'nucleus', 'positive regulation of DNA-templated transcription', 'regulation of DNA-templated transcription', and 'nucleolus'. The term 'intracellular membrane-bounded organelle' refers to organelles within the cell that are surrounded by membranes, including the nucleus, mitochondria, endoplasmic reticulum, Golgi apparatus, and lysosomes [18]. These findings suggest that the All-ten genes are involved in numerous major organelles that collaborate to maintain the balance of material metabolism and energy supply within the cell and ensure the correct folding and transportation of proteins. The enrichment for 'nucleus' indicates that many of these proteins are localized in the nucleus. Combined with the results of the overall enrichment analysis, most of the functions of the All-ten genes seem to be related to the regulation of gene expression. Therefore, it can be hypothesized that these proteins ensure the accurate and stable expression of genes in the nucleus by regulating processes such as transcription, DNA replication, and repair [19]. Furthermore, we analyse the accurate prediction of these enriched terms by DeepMTC, as depicted in **Figure 6**A (right panel). DeepMTC achieves exceptionally high accuracy in predicting the five key enriched terms, particularly 'regulation of DNA-templated transcription', with an AUROC of 90.88%.

Proteins localized in the cytoplasm are enriched in several GO terms, including 'intracellular membrane-bounded organelle' and 'nucleus', which overlap with the All-ten results. Additionally, these genes are enriched in GO terms such as 'phosphorylation', 'endosome membrane', and 'early endosome', as shown in **Figure 6**B (left panel). We find that some of the enriched GO terms are associated with endosomes, including 'endosome membrane' and 'early endosome', suggesting that the gene functions of these cytoplasm proteins are involved in endocytosis and are responsible for the transport and processing of substances within the cell [20]. The cytoplasm serves as the site for these biological processes and structures, ensuring normal cellular activity. Moreover, the enrichment results indicate involvement in partial gene function, regulation, cell signalling conduction, and apoptosis control, primarily within the nucleus. Proteins corresponding to these genes are located in the cytoplasm but are crucial for nuclear functions. These proteins are synthesized or activated in the cytoplasm and subsequently transported to the nucleus to regulate gene expression. Additionally, certain proteins move between the nucleus and cytoplasm during signalling processes. For example, upon phosphorylation and degradation of the IκB protein, the NF-kappaB dimer is released and translocates into the nucleus. There, it binds to specific DNA sequences, initiating gene transcription to regulate gene expression [21, 22]. Moreover, the prediction of key GO terms in the enrichment results for proteins localized in cytoplasm by DeepMTC is shown in **Figure 6**B (right panel), and the correct prediction of these GO terms by DeepMTC is remarkable. In particular, the AUROC predicted by DeepMTC for 'nucleus' is 73.23%. In conclusion, the results of the GO enrichment analysis described above highlight the close relationship between the subcellular localization of proteins and their functions.

**Supplementary Tables**

**Table S1.** Descriptive statistics of two species.

| Species(proteins) | GO | GO terms | Number of GO |
| --- | --- | --- | --- |
| Human (4127) | BP | 76 (119) | 345 |
|  | CC | 75 (101) | 6131 |
|  | MF | 55 (69) | 2362 |
| Mouse (1956) | BP | 119 (119) | 1965 |
|  | CC | 100 (101) | 3090 |
|  | MF | 69 (69) | 1705 |

**Table S2.** Subcellular localization and distribution of proteins.

| Subset | Subcellular location | Number of proteins |
| --- | --- | --- |
| S1 | Cell junction | 155 |
| S2 | Cell projection | 393 |
| S3 | Cell membrane | 2013 |
| S4 | Cytoplasm | 3011 |
| S5 | Lysosome | 152 |
| S6 | Secreted | 439 |
| S7 | Endoplasmic reticulum | 586 |
| S8 | Golgi apparatus | 444 |
| S9 | Mitochondrion | 692 |
| S10 | Nucleus | 2720 |
| Total different locative proteins | | 10605 |
| Total different proteins | | 6083 |

**Table S3.** Hyperparameter setting of DeepMTC.

| Hyperparameters | Setting |
| --- | --- |
| Number of GT layers | [1, **2**, 3, 4, 5] |
| Number of attention heads in GT | [2, 4, 6, **8**, 10] |
| Dimension of initial feature | [128, 256, **512**, 1024] |
| Pooling ratio (Subcellular localization) | [0.1, 0.2, 0.3, **0.4**, 0.5, 0.6, 0.7, 0.8, 0.9] |
| Number of encoder layers in Gae_block | [1, **2**, 3, 4] |
| Number of Fun_attention block attention heads | [2, **4**, 6, 8] |
| Batchsize | 20 |
| Epoch | 50 |

**Table S4.** Predicted GO terms of P01899 in MF by DeepMTC and competing methods.

| Methods | Go terms |
| --- | --- |
| PredGO | **GO:0042277**, **GO:0005515**, GO:0033218, |
| SPROF-GO | **GO:0005515**, GO:0033218, **GO:0042277** |
| NetGO 3.0 | **GO:0042277,** GO:0033218, **GO:0005515**, **GO:0042605**, **GO:0044877**, **GO:0030881**, **GO:0005102**, GO:0004888, GO:0038023, GO:0060089, GO:0046977 |
| DeepGOPlus | / |
| DeepFRI | / |
| GPSFun | **GO:0042277**, **GO:0005515** |
| DeepMTC | **GO:0042605, GO:0005102, GO:0030881, GO:0044877, GO:0042277** |
| Experimental  annotation | GO:0044877, GO:0042277, GO:0005102^η^, GO:0042605^ζ, η^, GO:0030881^ζ, η^, GO:0005515 |

Note: The correctly predicted GO terms are shown in bold. Terms that do not appear in PredGO, SPROF-GO and GPSFun are denoted by ζ, and terms that do not appear in DeepFRI are denoted by η.

**Table S5.** The ablation experiment result on the subcellular localization task.

| Method | AP | AUROC | AUPR | Acc | Fmax | Hloss (↓) | RL (↓) | Oerror (↓) |
| --- | --- | --- | --- | --- | --- | --- | --- | --- |
| DeepMTC w/o GT | 0.4546 | 0.9131 | 0.8320 | 0.9021 | 0.8174 | 0.0978 | 0.6746 | 0.1505 |
| DeepMTC w/o FunA | 0.6924 | 0.9157 | 0.8524 | 0.9069 | 0.8296 | 0.0930 | 0.4908 | 0.1271 |
| DeepMTC w/o FeaE | 0.5386 | 0.8871 | 0.8090 | 0.9009 | 0.8163 | 0.0990 | 0.5646 | 0.1648 |
| DeepMTC | 0.7841 | 0.9034 | 0.8432 | 0.9112 | 0.8349 | 0.0888 | 0.5064 | 0.1251 |

**Table S6.** The ablation experiment result on the functional prediction task.

|  |  | DeepMTC w/o GT | DeepMTC w/o FunA | DeepMTC w/o FeaE | DeepMTC |
| --- | --- | --- | --- | --- | --- |
| AUROC | BP | 0.6589 | 0.8020 | 0.8371 | 0.8484 |
|  | CC | 0.8645 | 0.8897 | 0.9066 | 0.9120 |
|  | MF | 0.8682 | 0.9212 | 0.9429 | 0.9483 |
| Fmax | BP | 0.6663 | 0.5684 | 0.6061 | 0.6498 |
|  | CC | 0.6649 | 0.6029 | 0.6441 | 0.6531 |
|  | MF | 0.6656 | 0.6579 | 0.6546 | 0.6704 |
| Macro_recall | BP | 0.5147 | 0.5851 | 0.5931 | 0.6428 |
|  | CC | 0.6529 | 0.6213 | 0.6511 | 0.6647 |
|  | MF | 0.6926 | 0.6874 | 0.6959 | 0.7203 |
| Macro_precision | BP | 0.5115 | 0.5556 | 0.5716 | 0.6007 |
|  | CC | 0.6443 | 0.6658 | 0.6708 | 0.6872 |
|  | MF | 0.7278 | 0.7386 | 0.7203 | 0.7570 |

**Table S7.** The impact of residue map construction threshold on the subcellular localization task.

| Thresholds (Å) | AP | AUROC | AUPR | Acc | Fmax | Hloss (↓) | RL (↓) | Oerror (↓) |
| --- | --- | --- | --- | --- | --- | --- | --- | --- |
| 10 | 0.7255 | 0.9145 | 0.8436 | 0.9095 | 0.8361 | 0.0904 | 0.4632 | 0.1437 |
| 8 | 0.7019 | 0.9150 | 0.8467 | 0.9120 | 0.8393 | 0.0880 | 0.4615 | 0.1291 |
| 4.5 | 0.7841 | 0.9034 | 0.8432 | 0.9112 | 0.8349 | 0.0888 | 0.5064 | 0.1251 |

**Table S8.** The impact of residue map construction threshold on the functional prediction task.

| Thresholds (Å) |  | 10 | 8 | 4.5 |
| --- | --- | --- | --- | --- |
| AUROC | BP | 0.8413 | 0.8336 | 0.8484 |
|  | CC | 0.9110 | 0.9138 | 0.9120 |
|  | MF | 0.9442 | 0.9475 | 0.9483 |
| Fmax | BP | 0.6959 | 0.7010 | 0.6498 |
|  | CC | 0.6574 | 0.6595 | 0.6531 |
|  | MF | 0.6813 | 0.7111 | 0.6704 |
| Macro_recall | BP | 0.7158 | 0.7780 | 0.6428 |
|  | CC | 0.6629 | 0.6947 | 0.6647 |
|  | MF | 0.7337 | 0.7371 | 0.7203 |
| Macro_precision | BP | 0.6059 | 0.6141 | 0.6007 |
|  | CC | 0.6837 | 0.6789 | 0.6872 |
|  | MF | 0.7770 | 0.7868 | 0.7570 |

**Supplementary Algorithm**

**Algorithm S1:** Algorithm of DeepMTC.

| **Algorithm S1:** Algorithm of DeepMTC. |
| --- |
| **Input:** Sequences of protein; |
| 1: Extract 3D structure of the sequences using ESMfold; |
| 2: Construct the residue graph $G_{\text{Res}}$ using predicted 3D structure; |
| 3: Extract the initial feature $H$ of the residues using ESM-2 and manually construct initial |
| feature of Edge; |
| 4: **for** *i* = 1→ *Epoch* **do** |
| 5: **for** *n* = 1 → *L* **do** |
| 6: Calculate the *l-*layer node feature $H_{p}^{l}$ and edge feature $E_{p}^{l}$for the input |
| sequence according to the (*l-*1) layer residue feature and edge feature using Eq. (1); |
| 7: **end for** |
| 8: Learning different functional features $H_{p}^{bp}$,$H_{p}^{cc}$ and $H_{p}^{mf}$ using Eq. (2); |
| 9: Prediction of protein function using the self-attention pooling mechanism of Eq. (3); |
| 10: Fusion of protein functional features using the functional cross-attention mechanism of Eq. (4); |
| 11: Calculate the multitask collaborative training loss using Eq. (27); |
| 12: Prediction of protein subcellular localization using Eq. (5) self-attention pooling mechanism and multilayer perceptron. |
| 14: **end for** |
| **Output:** a. Predicted protein function; b. Predicted protein subcellular localization. |

**Reference**

1. Kipf TN, Welling M. Semi-Supervised Classification with Graph Convolutional Networks. arXiv (Cornell University) 2016.
2. Davis J, Goadrich M. The relationship between Precision-Recall and ROC curves. Proceedings of the 23rd international conference on Machine learning - ICML ’06 2006.
3. Clark WT, Radivojac P. Information-theoretic evaluation of predicted ontological annotations. Bioinformatics 2013; 29:i53–i61.
4. Jiang Y, Oron TR, Clark WT, et al. An expanded evaluation of protein function prediction methods shows an improvement in accuracy. Genome Biology 2016; 17.
5. Zhang M-L, Zhou Z-H. A Review on Multi-Label Learning Algorithms. IEEE Transactions on Knowledge and Data Engineering 2014; 26:1819–1837.
6. Bromberg J, Darnell JE. The role of STATs in transcriptional control and their impact on cellular function. Oncogene 2000; 19:2468–2473.
7. Akiyama T. Wnt/β-catenin signaling. Cytokine & Growth Factor Reviews 2000; 11:273–282.
8. Webb AE, Brunet A. FOXO transcription factors: key regulators of cellular quality control. Trends in Biochemical Sciences 2014; 39:159–169.
9. Thumuluri V, Almagro Armenteros JJ, Johansen A, et al. DeepLoc 2.0: multi-label subcellular localization prediction using protein language models. Nucleic Acids Research 2022; 50:W228–W234.
10. Yuan Q, Tian C, Song Y, et al. GPSFun: geometry-aware protein sequence function predictions with language models. Nucleic acids research 2024; 52:W248–W255.
11. Zheng R, Huang Z, Deng L. Large-scale predicting protein functions through heterogeneous feature fusion. Briefings in bioinformatics 2023; 24.
12. Yuan Q, Xie J, Xie J, et al. Fast and accurate protein function prediction from sequence through pretrained language model and homology-based label diffusion. Briefings in bioinformatics 2023; 24.
13. Wang S, You R, Liu Y, et al. NetGO 3.0: Protein Language Model Improves Large-Scale Functional Annotations. Genomics, Proteomics & Bioinformatics/Genomics, proteomics and bioinformatics 2023; 21:349–358.
14. Kulmanov M, Hoehndorf R. DeepGOPlus: Improved protein function prediction from sequence. Bioinformatics 2019; 36:422–429.
15. Gligorijević V, Renfrew PD, Kosciolek T, et al. Structure-based protein function prediction using graph convolutional networks. Nature Communications 2021; 12.
16. Boqiao L, Jinbo X. Accurate protein function prediction via graph attention networks with predicted structure information. Briefings In Bioinformatics 2021; 23.
17. Jiao P, Wang B, Wang X, et al. Struct2GO: protein function prediction based on graph pooling algorithm and AlphaFold2 structure information. Bioinformatics 2023; 39.
18. Bozdech MJ, Bainton DF. Identification of alpha-naphthyl butyrate esterase as a plasma membrane ectoenzyme of monocytes and as a discrete intracellular membrane-bounded organelle in lymphocytes. Journal of Experimental Medicine 1981; 153:182–195.
19. Groth A, Rocha W, Verreault A, et al. Chromatin Challenges during DNA Replication and Repair. Cell 2007; 128:721–733.
20. Doherty GJ, McMahon HT. Mechanisms of Endocytosis. Annual Review of Biochemistry 2009; 78:857–902.
21. Xiao W. Advances in NF-kappaB signaling transduction and transcription. PubMed 2004; 1:425–35.
22. Yamauchi S, Ito H, Miyajima A. IκBη, a nuclear IκB protein, positively regulates the NF-κB–mediated expression of proinflammatory cytokines. Proceedings of the National Academy of Sciences 2010; 107:11924–11929.
